# Portals to frailty? Data-driven analyses detect early frailty profiles

**DOI:** 10.1186/s13195-020-00736-w

**Published:** 2021-01-04

**Authors:** Linzy Bohn, Yao Zheng, G. Peggy McFall, Roger A. Dixon

**Affiliations:** 1 Department of Psychology, University of Alberta, P217 Biological Sciences Building, Edmonton, AB T6G 2E9 Canada; 2 Neuroscience and Mental Health Institute, University of Alberta, 2-132 Li Ka Shing Center for Health Research Innovation, Edmonton, AB T6G 2E1 Canada

**Keywords:** Latent profile analysis, Frailty, Neurocognitive speed, Victoria longitudinal study, Trajectories

## Abstract

**Background:**

Frailty is an aging condition that reflects multisystem decline and an increased risk for adverse outcomes, including differential cognitive decline and impairment. Two prominent approaches for measuring frailty are the frailty phenotype and the frailty index. We explored a complementary data-driven approach for frailty assessment that could detect early frailty profiles (or subtypes) in relatively healthy older adults. Specifically, we tested whether (1) modalities of early frailty profiles could be empirically determined, (2) the extracted profiles were differentially related to longitudinal cognitive decline, and (3) the profile and prediction patterns were robust for males and females.

**Methods:**

Participants (*n* = 649; *M* age = 70.61, range 53–95) were community-dwelling older adults from the Victoria Longitudinal Study who contributed data for baseline multi-morbidity assessment and longitudinal cognitive trajectory analyses. An exploratory factor analysis on 50 multi-morbidity items produced 7 separable health domains. The proportion of deficits in each domain was calculated and used as continuous indicators in a data-driven latent profile analysis (LPA). We subsequently examined how frailty profiles related to the level and rate of change in a latent neurocognitive speed variable.

**Results:**

LPA results distinguished three profiles: *not-clinically-frail* (NCF; characterized by limited impairment across indicators; 84%), *mobility-type frailty* (MTF; characterized by impaired mobility function; 9%), and *respiratory-type frailty* (RTF; characterized by impaired respiratory function; 7%). These profiles showed differential neurocognitive slowing, such that MTF was associated with the steepest decline, followed by RTF, and then NCF. The baseline frailty index scores were the highest for MTF and RTF and increased over time. All observations were robust across sex.

**Conclusions:**

A data-driven approach to early frailty assessment detected differentiable profiles that may be characterized as morbidity-intensive portals into broader and chronic frailty. Early inventions targeting mobility or respiratory deficits may have positive downstream effects on frailty progression and cognitive decline.

**Supplementary Information:**

The online version contains supplementary material available at 10.1186/s13195-020-00736-w.

## Background

Frailty is a heterogeneous condition that reflects accumulated age-related multi-morbidity, leading to diminished physical function and reduced physiological reserve [1]. Progression along the fitness-frailty continuum is associated with an increased risk for numerous adverse aging outcomes [2], including differential cognitive decline, impairment, and dementia [3–5]. Against this backdrop, frailty is characterized as the most problematic expression of population aging [6] and has been established as a priority area in clinical and research settings [7]. Yet, considerable debate continues regarding the measurement and conceptualization of frailty. At present, two productive approaches dominate the literature: the physical frailty phenotype [8] and the frailty index [9]. We explored a third approach which could be applicable to early detection of elevated frailty risk: data-driven frailty assessment.

The physical phenotype approach defines frailty using the following cluster of variables: unintentional weight loss, self-reported exhaustion, weak grip strength, slow gait, and low physical activity. Notably, the phenotypes are ordered on the basis of the number of deficits, such that an individual with no deficits is classified as robust, one to two deficits is pre-frail, and three or more deficits is frail [6]. Because this approach incorporates a restricted number of physical characteristics, it may be limited in early detection of frailty risk. In contrast, the frailty index embraces heterogeneity in that responses across multiple indicators of aging systems are summed to create a single score that represents the ratio of deficits present in an individual relative to the total number of deficits considered. However, values on the index reflect the number of deficits that an individual has accumulated—and in pre-clinical aging, the global frailty index may be relatively low while specific morbidity sources or domains of impairment are emerging. New data-driven analytic technologies may be useful in early detection of frailty profiles that serve as portals to the emergence of global frailty in aging—and as harbingers of a host of adverse aging outcomes.

Accordingly, we applied latent profile analysis (LPA) to a database of multi-morbidity indicators in order to detect underlying clusters or profiles of early frailty. LPA is a data-driven, person-centered statistical approach that can identify homogenous subgroups of individuals based on a set of observed indicators [10]. This statistical approach is analogous to latent class analysis (LCA)—with the exception that the indicators are continuous. LPA is a sensitive analytical technique for studying heterogeneous clinical syndromes for which there is limited consensus on its defining and emergent characteristics [11]. Findings from this study will advance the literature on measurement, analysis, and conceptualization of frailty by identifying empirically derived frailty profiles that are not differentiated on the basis of the number of physical impairments or proportion of accumulated deficits. Instead, detected profiles would reflect empirically observed classes of deficits, within a broad spectrum of morbidity, sharing pattern, and severity characteristics.

Interestingly, identification of clusters of vulnerabilities, signs, and symptoms of frailty was established as a priority area in the beginning stages of this field [12, 13]. Some experts reasoned that detection of frailty subtypes may contribute to a refined definition that would be useful for understanding the antecedents, emergence, or differential mechanisms associated with the variety of deficits subsumed under this general construct. Nevertheless, few studies have employed data-driven statistical techniques to distinguish frailty profiles based on multidomain deficit accumulation. Recently, Sadiq and colleagues [14] assembled 18 items related to physical, functional, emotional, and social deficits and subjected these data to an LCA. Findings revealed three discrete frailty profiles that differed primarily in overall severity: (a) *not frail*, which was characterized by minimal impairment across all morbidity indicators, (b) *moderately frail*, which was characterized by moderate physical and functional limitations, and (c) *severely frail*, which was characterized by severe limitations in physical, functional, and emotional health. These findings converge with an earlier study that subjected 41 items related to self-reported health, cognitive function, social function, mental health, morbidity status, and functional limitations to an LCA [15]. The following six frailty profiles, that also differed primarily in overall severity, were distinguished: *relatively healthy*, *mild physically frail*, *psychologically frail*, *severe physically frail*, *medically frail*, and *multi-frail*. The relatively healthy profile was characterized by minor problems across all indicators, whereas the remaining profiles were characterized by singular deficits in either physical or psychological health (at varying levels of severity), or by a combination of physical, psychological, cognitive, and social deficits.

We extend this prior work by determining which frailty profiles representing distinct configurations of aging morbidity are detected and examining how they are related to level and change trajectories in neurocognitive speed. Accumulating literature suggests that frailty and cognitive impairment are related but distinct concepts that frequently co-occur in older age [16, 17]. Yet, few studies have examined broader definitions of frailty in relation to normal age-related decline in specific domains of cognition [18], such as neurocognitive speed [19–21]. Given that non-memory domains may be particularly susceptible to early frailty effects [22], this is an important target of research attention. Findings from this study may advance understanding of whether there are specific combinations of deficits that appear early on in the frailty trajectory that predict an increased risk for accelerated cognitive decline.

Data were drawn from the Victoria Longitudinal Study (VLS), which is a multi-faceted, large-scale, long-term investigation of biomedical and neurocognitive aging [23]. We assembled baseline data for each participant that included 50 items representing the typical heterogeneity of frailty [4]. We used exploratory factor analysis to reduce the total number of items for estimation feasibility in the LPA. These results produced separable health domains that were interpreted on the basis of previous research [14, 24, 25]. The proportion of deficits accumulated in each domain was calculated for each participant and used as continuous observed indicators in the LPA.

A recent VLS study used these same 50 items to calculate a frailty index and investigated whether the level and/or rate of change in frailty predicted performance and decline in neurocognitive speed across a 40-year band of aging [4]. Findings showed that the level of frailty at baseline was predictive of neurocognitive speed performance at baseline. Moreover, change in the level of frailty was related to the rate of change in neurocognitive speed performance. Of note, these effects were moderated by sex, such that frailty change predicted the change in speed selectively for females, whereas frailty was unrelated to level or change in speed for males. At least one other study pointed to sex differences in the mechanisms linking frailty with early changes in cognitive function [26]. Given these findings and those from related research [27, 28], we tested whether our results were robust across sex.

The specific research goals (RG) of this study were as follows. For RG1, we employed LPA in order to detect empirically derived frailty profiles. As the sample was relatively healthy, we expected to observe early frailty profiles that differed in the nature of deficit accumulation. For RG2, we investigated how frailty profiles related to performance and decline in neurocognitive speed. We expected to observe frailty-cognition associations, although the extent could vary across detected profiles. For RG3, we tested whether profile membership and trajectory predictions generalized across sex.

## Methods

### Participants

Participants were community dwelling older adults from the VLS who provided written and informed consent. Both the VLS and data collection procedures were in full and certified compliance with prevailing human research ethics guidelines and boards. The VLS is comprised of longitudinal cohorts that were aged 53–85 years at recruitment. Continuing participants were tested at an average of 4.4-year intervals. The source cohort for this study (*n* = 693) provided (a) baseline multi-morbidity data and (b) three waves of neurocognitive speed data. In accordance with established procedures for accelerated longitudinal designs [29, 30], age was used as the metric of longitudinal change. This approach allowed us to control for age-related effects and increase interpretability of the findings. The resulting design spans a 40-year band of aging [28].

The following exclusionary criteria were applied at baseline: (a) diagnosis of Alzheimer’s or dementia (*n* = 0), (b) missing data across each of the 50 multi-morbidity items at baseline (*n* = 40), and (c) missing data across all waves and indicators of the latent speed variable (*n* = 4). Descriptive statistics for the remaining sample are outlined in Table 1 (*n* = 649; 431 females; *M* age = 70.61, SD = 8.64, age range = 53–95 years; primarily White). Retention rates were 82% for wave 1 to wave 2 and 78% for wave 2 to wave 3.

**Table 1:** Participant characteristics at baseline

Characteristic	Total sample	Not-clinically-frail	Mobility-type	Respiratory-type	Sig.
Class prevalence *n* (%)	–	542 (84%)	59 (9%)	48 (7%)	
*n* (%) female	431 (66%)	351 (65%)	44 (75%)	36 (75%)	*ns*
Age (in years)	70.61 (8.64)	69.78 (8.39)^e^	78.21 (7.53)^f^	70.60 (8.27)^e^	***
Education (in years)	15.27 (2.97)	15.39 (2.94)	14.67 (2.83)	14.54 (3.34)	*ns*
*APOE* ɛ4+	150	132 (24%)	6 (11%)	12 (25%)	*ns*
Frailty index^a^	0.13 (0.07)	0.11 (0.06)^e^	0.22 (0.07)^f^	0.20 (0.07)^f^	***
MMSE	28.67 (1.25)	28.70 (1.24)	28.29 (1.39)	28.78 (1.11)	*ns*
Timed walk^b,c^	6.42 (1.65)	6.12 (1.13)^e^	9.28 (2.80)^f^	6.51 (1.65)^e^	***
Peak flow (L/min)^b,d^	421.98 (117.77)	435.40 (114.01)^e^	360.17 (100.86)^f^	329.10 (123.31)^g^	***

Results presented as mean (standard deviation). *p* values are based on one-way ANOVA or chi-square tests, as appropriate. We adjusted for multiple comparisons using post-hoc Tukey tests

^a^We calculated the proportion of deficits for each person on the 50 item frailty index as reported in Thibeau et al. [4]

^b^We tested whether mobility- and respiratory-type differed from one another and the not-clinically-frail profile using planned comparisons

^c^The number of seconds taken to walk 20 ft

^d^The largest volume of air expired over three attempts

^e, f, g^Values with different superscripts differ from one another

***p < .001

### Measures

#### Multi-morbidity data

We assembled baseline data for 50 multi-morbidity items that (a) have been used in the VLS and related research to form a frailty index [4], (b) have demonstrated associations with adverse brain and cognitive aging outcomes [4], and (c) satisfy prevailing conventions surrounding deficit accumulation approaches to frailty assessment [31]. Data for these items were collected using self-report, physical examinations, and formal tests with standardized scales. All items were recoded such that scores ranged from 0 (no deficit present) to 1 (deficit was maximally expressed [31]; see Table 2 for examples; full list in Supplementary Table 1, Additional File 1).

**Table 2:** Multi-morbidity items by exploratory factor analysis derived frailty domain

Domain	Indictor
Mobility	Finger dexterity^a^
	Timed turn^a^
	Grip strength^b^
	Use of walker, cane, or wheelchair^c^
Instrumental health	Health has affected ability to travel^d^
	Health has affected ability to socialize^d^
	Health has affected ability to do hobbies^d^
	Health has affected ability to do mental activities^d^
	Health has affected ability to get around town^d^
	Health has affected ability to do chores^d^
Emotional wellbeing	Bradburn negative affect (restless, lonely, bored, depressed, upset due to criticism)^e^
	CES-D “during the past week, my sleep was restless”^f^
	CES-D “during the past week, I felt depressed”^f^
	CES-D “during the past week, I felt lonely”^f^
Comorbidity	Anemia^g^
	Sex-related health problems (i.e., gynecological problems or prostate problems)^g^
	Gastrointestinal problems (colitis/diverticulitis, gall bladder trouble, and/or liver trouble)^g^
	Kidney or bladder trouble^g^
Respiratory symptoms	Feeling short of breath^c^
	Bronchitis or emphysema^g^
	Asthma^g^
Cardiac symptoms	Pulse pressure^h^
	Heart trouble^g^
	Hardening of arteries (i.e., atherosclerosis)^g^
	High blood pressure^g^
	Stroke^g^
Physical activity	Stay at home but in chair most of the time^c^
	Health has affected ability to do physical recreational activities^d^
	Spinal condition and/or back trouble^g^
	Arthritis (rheumatoid and/or osteo)^g^

^a^Performance was recoded as 0 (< 90th percentile) or 1 (within 90th percentile)

^b^Performance was recoded as 0 or 1. See Supplementary Table 1, Additional File 1

^c^0 = no, 1 = yes

^d^0 = no change, improved, N/A; 0.25 = slightly reduced; 0.50 = moderately reduced; 0.75 = drastically reduced; 1 = gave up doing activity

^e^0 = no to all; 0.2 = yes to one; 0.4 = yes to two; 0.6 = yes to three; 0.8 = yes to four; 1 = yes to all

^f^0 = rarely or none of the time; 0.33 = some or a little of the time; 0.67 = occasionally or a moderate amount of the time; 1 = most or all of the time

^g^0 = no; 0.33 = yes, not serious; 0.67 = yes, moderately serious; 1 = yes, very serious

^h^Performance was recoded as 0 = 32.13–63.90; 0.5 = 64–75.9; 1 = 76+

#### Neurocognitive speed

We represented neurocognitive speed as a multi-indicator latent variable using the following four manifest indicators: simple reaction time, choice reaction time, lexical decision, and sentence verification. Each of these indicators are multi-trial, computer-based neuropsychological tasks that have (a) established psychometric properties, (b) been widely used and documented in the VLS and related cognitive aging research, and (c) demonstrated sensitivity to neurocognitive factors and functional biomarkers [4, 32]. The target measure for each task was the average response latency across the test trials. Responses were recoded such that higher scores represented better performance. We present descriptions of each task and data correction procedures in the Supplementary Methods, Additional File 1.

### Statistical analyses

Analyses were conducted using Mplus 8.0 [33]. Missing data were handled using full information maximum likelihood unless specified as otherwise.

#### Foundational analyses

The following foundational analyses served the purpose of testing and confirming basic characteristics of the neurocognitive speed data, as well as preparing the latent variable: (a) confirmatory factor analysis, (b) longitudinal measurement invariance tests, and (c) unconditional latent growth modeling. Further details are presented in the Supplementary Methods, Additional File 1.

#### Focal analyses

The 50 multi-morbidity items were submitted to an exploratory factor analysis. Importantly, we made decisions related to the number of factors (health domains) and which indicators to retain on the basis of best-practices literature [34, 35]. We verified that this latent structure fit the data using confirmatory factor analysis. Model fit was determined using standard indices (see Supplementary Methods, Additional File 1).

For the latent profile analysis (LPA), we fit a sequence of models with varying numbers of latent profiles (e.g., 1, 2, 3). We selected the best fitting model based on interpretability of the study findings, as well as the following model parameters, tests, and fit indices [36]: (a) log-likelihood value (*LL*), (b) number of parameters estimated, (c) Bayesian Information Criterion (BIC), (d) sample-size adjusted BIC (SABIC), (e) Akaike Information Criterion (AIC), (f) adjusted Lo-Mendell-Rubin likelihood ratio test (LMR-LRT), (g) adjusted Vuong-Lo-Mendell-Rubin likelihood ratio test (VLMR-LRT), and (h) entropy. Low values of BIC, SABIC, and AIC indicate better fit [10]. The LMR-LRT and VLMR-LRT compare the current model (*k*) against the model of one fewer latent profile (*k-1*); a non-significant *p* value supports the selection of the *k-1* profile model [10]. Entropy (ranging between 0 and 1) is not used for model selection but suggests the classification accuracy (the higher the better).

To avoid local maxima, we used 5000 multiple starting values. Indicators were allowed to covary within class, while the variances-covariances were constrained to be equal across profiles (i.e., class invariant-unrestricted structure). Alternative models allowing free estimation of variance-covariance across profiles did not converge, suggesting over-parameterization [37]. We controlled for potential age effects by regressing the observed indicators and profile membership on age. An adapted formula for Cohen’s *d* was used to (a) calculate standardized mean differences across latent profiles in the observed indicators and (b) facilitate interpretations of the final latent-profile solution [36]. Values > 2.0 indicate a less than 20% overlap in profile-specific distributions and a high degree of separation on the associated indicator, whereas values < 0.85 indicate more than 50% overlap and a low degree of separation on the associated indicator.

We examined how the frailty profiles related to intercept (performance at a statistical centering age) and linear slope (longitudinal change) of neurocognitive speed using the manual BCH method (for further details, see [38, 39]). We tested whether latent profiles differed in the level or rate of change by comparing the nested models with constrained equal performance level (i.e., intercept) or decline in speed (i.e., linear slope) with the full model where performance level and decline in speed were freely estimated for each latent profile using *χ*^2^ tests. Significant differences were inferred from a -2*LL* difference statistic (*D* at *p* < .10), which compared the unconstrained model to the constrained model.

We tested whether membership in the frailty profiles was comparable across sex by performing a multinomial logistic regression using the R3step approach (for further details, see [40]). We examined whether frailty-cognition associations generalized across sex by regressing the intercept and slope of speed on sex separately for each profile.

## Results

### Foundational analyses

Results of the confirmatory factor analysis indicated that a single-factor latent variable model for neurocognitive speed fit the data adequately. Measurement invariance tests showed full metric and full scalar invariance (final model fit indices: root mean square error of approximation (RMSEA) = .08; comparative fit index (CFI) = .96; standardized root mean square residual (SRMR) = .09; see Supplementary Table 2, Additional File 1). Regarding the latent growth model for speed, participants demonstrated (a) significant variation in level of performance ($\hat{\upsigma}$ ^2^ = 1.00, *p* < .001), (b) significant decline over time (*M* = −.074, *p* < .001), and (c) significant interindividual differences in the rate of decline ($\hat{\upsigma}$ ^2^ = .003, *p* < .001; see Supplementary Table 3, Additional File 1). This model was subsequently used to generate intercept and linear slope estimates for each participant, which then served as the target distal outcome measures.

### RG1a: Exploratory and confirmatory factor analysis for multi-morbidity items

Results from the exploratory factor analysis indicated that a 7-factor solution adequately explained associations amongst the final 30 multi-morbidity items. We tested whether this latent structure fit the study data using confirmatory factor analysis. Results showed adequate to good model fit (*χ*^2^(384) = 649.02, *p* < .001; RMSEA = .03; CFI = .90) and all indicators had strong loadings on the corresponding latent construct (for model depiction see Supplementary Fig. 1, Additional File 1). In accordance with earlier research [14, 24, 25], we labeled these domains as: mobility (*n =* 4), instrumental health (*n* = 6), emotional wellbeing (*n* = 4), comorbidity (*n* = 4), respiratory symptoms (*n* = 3), cardiac symptoms (*n* = 5), and physical activity (*n* = 4). Indicators for each domain are outlined in Table 2. We subsequently calculated the proportion of deficits in each domain for each participant. Values ranged between 0 and 1, with higher scores denoting greater impairment. These data were used as continuous observed indicators in the LPA.

### RG1b: Identification of latent frailty profiles

As shown in Table 3, AIC, BIC, and SABIC all steadily decreased (i.e., became more negative) as the number of latent profiles increased, suggesting that model fit improved with the addition of each latent profile. Further, the adjusted LMR-LRT (*p* < .001) and VLMR-LRT (*p* < .001) indicated that the 3-profile solution provided better fit relative to the 2-profile solution. Notably, prevalence of each profile exceeded a conventional standard of 5% [10]. Entropy for this solution was also high (0.99), indicating that participants were classified into the profiles with a high degree of precision. The 3-profile solution was therefore selected as the final model.

**Table 3:** Model fit indices for one- to four-latent profile solutions

Profile	(−)2LL	npar	AIC	BIC	SABIC	LMR	VLMR	Entropy
1	− 5029.43	42	− 4945.43	− 4757.47	− 4890.81	–	–	–
2	− 5584.96	51	− 5482.96	− 5254.72	− 5416.64	< .001	< .001	0.99
3	**− 5880.55**	**60**	**− 5760.55**	**− 5492.02**	**− 5682.52**	**< .001**	**< .001**	**0.99**
4^b^	− 6235.52	69	–	–	–	–	–	–

*(−2)LL* − 2 log-likelihood; *npar* number of parameters free; *AIC* Akaike information criterion; *BIC* Bayesian information criterion; *SABIC* sample size adjusted BIC; *LMR* adjusted Lo-Mendell-Rubin likelihood ratio test; *VLMR* adjusted Vuong-Lo-Mendell-Rubin likelihood ratio test

^b^This model was not considered due to non-replicated log-likelihood

#### Interpretation of the frailty profiles

**Fig. 1:**
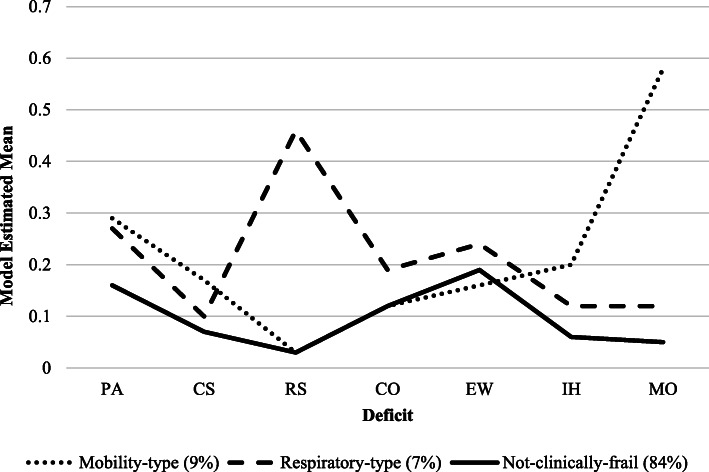
Model estimated observed indicator means for each latent profile. PA, physical activity; CS, cardiac symptoms; RS, respiratory symptoms; CO, comorbidity; EW, emotional well-being; IH, instrumental health; MO, mobility. For further explanation of the profile interpretations, see the “Results” section

Model estimated indicator means for each latent profile are depicted in Fig. 1. The first profile (*n* = 542, 84%) was characterized by relatively low impairment across all observed indicators and was thus labeled as *not-clinically-frail* (NCF). Notably, participants in this profile had an average score on the frailty index (see Table 1) that fell below the clinical threshold typically used to assign frailty status, whereas the remaining two profiles had scores that met or exceeded a previously established cutoff value of .20 [31]. The second profile (*n* = 59, 9%) was characterized by pronounced impairment in mobility function relative to the first (*d* = 5.09) and third (*d* = 3.90) profile. This profile was thus labeled as *mobility-type frailty* (MTF). The third profile (*n* = 48, 7%), labeled as *respiratory-type frailty* (RTF), was characterized by pronounced impairment in respiratory function relative to the NCF (*d* = 6.96) and MTF profiles (*d* = 4.72). Interestingly, none of these profiles were distinguished on the basis of emotional well-being, comorbidity, cardiac symptoms, or physical activity (for details see Supplementary Table 4, Additional File 1). As highlighted in Table 1, the pattern of mean differences observed across profiles in performance-based tasks was in keeping with our interpretations. That is, participants classified into MTF had the slowest performance on a timed-walk task, while participants classified into RTF had the lowest peak-expiratory flow. We present further descriptive baseline information for each latent profile in Table 1.

In a series of follow-up analyses, we tested whether the MTF and RTF profiles function as morbidity-intensive portals that subgroups of older adults pass through into classifiable chronic frailty. We assembled three waves of data for the 50 item frailty index [4] and calculated a growth model over the 40-year longitudinal band. Key results showed significant (a) variation in the level of frailty ($\hat{\upsigma}$ ^2^ = .004, *p* < .001), (b) increase in frailty over time (*M* = .003, *p* < .001), and (c) interindividual differences in the rate of frailty progression ($\hat{\upsigma}$ ^2^ = .001, *p* < .001; see Supplementary Table 3, Additional File 1). We generated intercept and linear slope estimates for each participant and tested whether the profiles were differentially related to level (severity) and rate of change in the frailty index using the manual BCH approach. Evidence in support of a portal approach to frailty emergence and progression would be constituted by a higher level and steeper rate of deficit accumulation for MTF and RTF as compared to the NCF profile.

#### Results for portal-related analyses

**Fig. 2:**
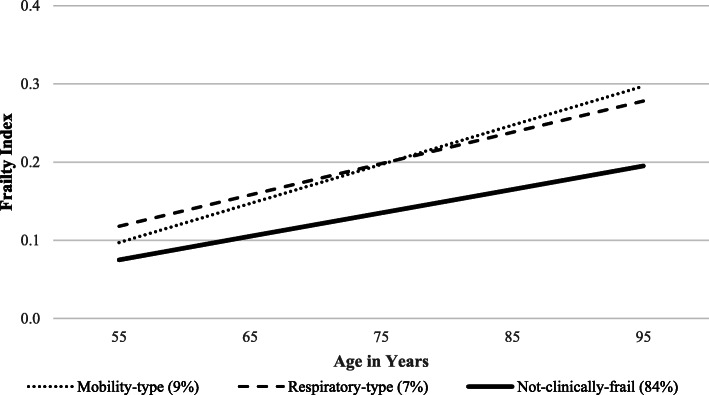
Predicted growth curve model for the 50 item frailty index across profile. Age in years was used as the metric of change and centered at 75 years. Profiles differed significantly in intercept and slope

The predicted growth curve model for the frailty index is presented in Fig. 2. Consistent with our expectations, profiles differed significantly in intercept. Specifically, older adults with MTF (*b* = .20, *p* < .001) and RTF (*b* = .20, *p* < .001) had higher (worse) scores on the frailty index relative to those who were NCF (*b* = .14, *p* < .001; *D* = 11.20, Δ*df* = 4, *p* < .001). Differences across profiles in the rate of frailty progression (slope) were also in the expected direction. MTF was associated with the fastest rate of deficit accumulation (*b* = .005, *p* < .001), followed in order by RTF (*b* = .004, *p* < .001; *D* = 8.62, Δ*df* = 2, *p* = .01), and then NCF (*b* = .003, *p* < .001; *D* = 14.71, Δ*df* = 2, *p* < .001).

### RG2: Latent profile-speed associations

**Fig. 3:**
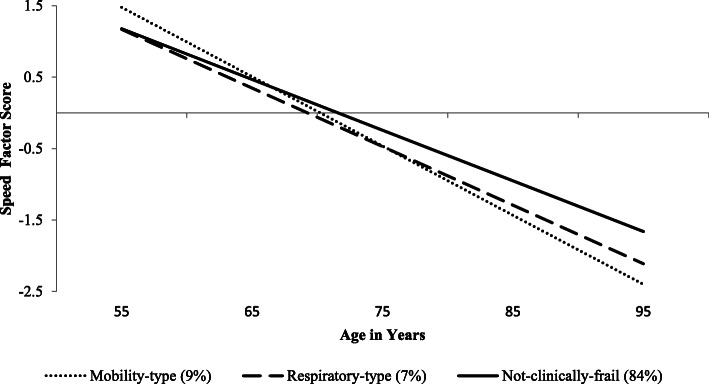
Predicted growth curve model for speed factor scores across profile. Age in years was used as the metric of change and centered at 75 years. Intercept was comparable across profiles. Slope differed significantly across profiles

The predicted growth curve model for neurocognitive speed is depicted in Fig. 3. Intercept did not vary significantly across MTF (*b* = −.46, *p* < .001), RTF (*b* = −.47, *p* < .001), and NCF (*b* = −.24, *p* < .001) profiles (*D* = 7.87, Δ*df* = 4, *p* = .10). However, we observed significant differences across profiles in the rate of cognitive decline (slope; *D* = 31.81, Δ*df* = 4, *p* < .001). Specifically, MTF (*b* = −.10, *p* < .001) was associated with more precipitous decline relative to RTF (*b* = −.08, *p* < .001; *D* = 13.90, Δ*df* = 2, *p* < .001) and the NCF profile (*b* = −.08, *p* < .001; *D* = 23.61, Δ*df* = 2, *p* < .001). RTF was also associated with more accelerated decline relative to the NCF profile (*D* = 7.88, Δ*df* = 2, *p* = .02).

### RG3: Generalizability of profile membership and prediction patterns across sex

We found that profile membership was similar across sex (coded as 0 = female, 1 = male) such that male sex was equally related to the likelihood of being classified into MTF (OR = .62, *ns*) or RTF (OR = 0.61, *ns*) as compared to NCF. Further, male sex was equally related to the likelihood of being classified into RTF as compared to MTF (OR = .98, *ns*). Similarly, sex showed comparable associations with the level and rate of change in neurocognitive speed for each of the frailty profiles (all *p* values > .20).

## Discussion

The frailty phenotype [8] and the frailty index [9] are the two important and productive approaches to measuring, conceptualizing, and investigating frailty. Each of these approaches has been widely used to capture variations in the risk for adverse aging outcomes [41], including accelerated cognitive decline and dementia [3, 5]. The present study examined a complementary approach that relied on data-driven statistical techniques. Specifically, we submitted 50 items to an exploratory factor analysis and derived the following 7 domains of aging morbidity: mobility, instrumental health, emotional wellbeing, comorbidity, respiratory symptoms, cardiac symptoms, and physical activity [14, 24, 25]. We calculated the proportion of deficits accumulated in each domain and submitted these data to a latent profile analysis (LPA) in order to detect frailty profiles. We then examined whether (a) distinguishable early frailty profiles could be empirically detected and characterized, (b) frailty profiles differentially predicted the level and rate of change in neurocognitive speed, and (c) profile membership and prediction of cognitive trajectories was comparable across sex.

### RG1: Identification of latent frailty profiles

In this person-centered analysis, three mutually exclusive early morbidity profiles of individuals were identified. The first profile we identified, *not-clinically-frail* (NCF), has been reliably documented in related research [14, 15, 42, 43] and was characterized by individuals with minimal impairment across the observed indicators and low scores on the frailty index. This pattern would be expected in a relatively healthy and cognitively normal aging group, and thus would include numerous persons who could later develop global or phenotypic frailty. The second profile, *mobility-type frailty* (MTF), was differentiated on the basis of deficits in mobility function. This profile is consistent with some research that suggests mobility deficits may aggregate to form a unique frailty subtype [44–46]. For example, Liu and colleagues [42] recently applied latent class analysis (LCA) to the five items from the physical frailty phenotype and detected four subtypes, one of which was labeled *mobility-type*. The present study extracts this subtype from a much broader range of morbidity measures and identifies it as an early frailty profile. The third profile we detected represented *respiratory-type frailty* (RTF). This profile was comprised of individuals with pronounced impairment in respiratory function. Identification of RTF as an early frailty profile advances the literature on subgroups of frail older adults. Although expanding [47], the vast majority of available works have conceptualized frailty using only the physical phenotype [42, 48–50] or have not included respiratory symptoms and diseases in the measurement of aging morbidity [14, 15]. However, another recent study also distinguished a data-driven frailty subtype marked by concomitant respiratory impairment [51]. Our results suggest that deficits in respiratory function are a defining characteristic of early frailty profiles and should be targeted and tracked in clinical and research settings [43, 52, 53]. Finally, we note that older adults classified into the two early frailty profiles had comparable scores on the 50 item frailty index—and these scores exceeded those of the non-frail group and met an established threshold for clinical frailty [31].

We tested whether MTF and RTF may represent early and specific morbidity-intensive portals into broader and chronic frailty in a series of follow-up analyses. Notably, the results buttressed this interpretation. Not only did older adults classified as MTF or RTF have higher levels of frailty (intercept), but they also showed more rapid progression into general frailty as compared to those who were NCF (slope). Interestingly, MTF was also associated with a faster rate of deficit accumulation as compared to RTF. These findings contribute to the emerging literature on trajectories of frailty [54] and extend earlier research that reported single indicators of mobility [55, 56] and respiratory function [57, 58] are predictive of frailty progression. We advance these works by proposing and validating a portal approach to frailty emergence, which reasons that profiles of aging morbidity marked by mobility or respiratory deficits may serve as gateways to classifiable global frailty, which then cascades into more rapid and widespread deficit accumulation [59]. The present focus on detecting early manifestations of frailty profiles and the representation of these as portals into global frailty is a promising research direction. Future epidemiological studies would profitably be directed towards replicating and extending these results (e.g., data-driven frailty assessment in clinical cohorts).

### RG2: Latent profile-speed associations

We found that, while the two emergent frailty profiles differed only marginally for prediction of level (intercept of neurocognitive speed), they differed significantly for slope (decline or slowing). Regarding level, the pattern of effects was in the expected direction [4, 19, 21]. Specifically, older adults classified into MTF or RTF subtypes trended towards worse performance relative to those who were NCF. Notably, regarding slope, older adults classified as having MTF showed the most precipitous decline, followed in order by RTF and then NCF. These relationships support the validity of these profiles and suggest that distinct configurations of aging morbidity marked by deficits in mobility and respiratory function may have differential effects on neurocognitive slowing. We note that these results cannot be attributed to age, educational background, or proportion of deficits accumulated. Three reasons are noted. First, we statistically controlled for the effects of age. Second, the frailty profiles did not differ from one another in their level of educational achievement. Third, participants assigned to MTF and RTF had comparable baseline scores (and intercept values) on the frailty index and yet they differed in the rate of decline.

To our knowledge, this is the first study to determine data-driven early frailty profiles using LPA and examine their prediction of cognitive aging trajectories. Of the related works summarized above, cognition was treated variably as (a) a study covariate [48], (b) amongst one of the indicators of aging morbidity [15, 49], or (c) not relevant or included in the analysis [14, 50]. Notably, Liu and colleagues [42] explored descriptive differences across frailty subtypes and reported findings that run in parallel to our own in mobility-type frailty was associated with lower scores on the MMSE relative to the robust subtype. Other research highlights that single indicators of mobility or physical function, such as gait speed or grip strength, are associated with decline in processing speed [60, 61]. Far less research has examined respiratory-cognition associations [62], particularly within the context of frailty [58]. Olaya and colleagues [43] recently reported that older adults assigned to a *cardiorespiratory* latent multi-morbidity profile had worse verbal memory performance relative to a *healthy* profile. Several recent reviews have also reported that single indicators of respiratory function, such as forced expiratory volume or asthma, predict neurocognitive slowing [62, 63]. Nevertheless, this is the first study to extract MTF and RTF profiles from a multi-morbidity inventory in mostly non-frail older adults and then systematically compare them in their initial frailty scores (similar), rate of frailty progression (dissimilar), and their predictions of cognitive change trajectories (dissimilar). These results suggest older adults presenting with deficits in mobility or respiratory function may be particularly vulnerable to advancing frailty and accelerated neurocognitive slowing. Proper assessment and management of these signs, symptoms, and diseases as they appear early on in the frailty trajectory is therefore encouraged. Accumulating literature suggests that frailty is a potentially reversible condition [64]. It has therefore been reasoned that early interventions designed to reverse or attenuate frailty progression may have downstream effects on reducing negative aging outcomes, including differential cognitive decline and impairment [16, 65].

### RG3: Generalizability of profile membership and prediction patterns across sex

Limited research has examined whether data-driven early frailty profiles, particularly those derived on the basis of multidomain deficit accumulation, are robust across sex. A small number of studies have explored whether the proportion of males and females assigned into frailty profiles is comparable; however, this question differs conceptually from the one tested in the present study and the earlier findings were equivocal [14, 42, 49]. Our results indicated that males and females were equally likely to be classified into the MTF, RTF, and NCF profiles. Looman and colleagues [15] also examined whether profile membership generalized across sex and reported findings that converge with our own. Previous literature suggests that there may be sex differences in the impact of frailty on cognitive aging trajectories [4, 5, 26]. However, we did not detect such a pattern in our data. Rather, we found that performance and decline in neurocognitive speed was comparable across sex. Sex differences may be more likely to appear in later life or in more serious frailty conditions.

Given the heterogeneity of frailty, the mechanisms underlying the observed associations are unclear. Current reviews attribute frailty-cognition associations to hormonal dysregulation, nutritional factors and deficiencies, chronic inflammation, and cardiovascular risks [16, 18, 66]. Perhaps more relevant for the present research are studies showing that non-demented older adults accumulate neuropathology [67–69] and show structural and functional declines [70] in the regions that underlie motor functions and processing speed, such as the striatum, substantia nigra, and motor cortices. Increased white matter hyper-intensities and decreased cerebellar gray matter volumes have also been linked with reduced mobility function [46] and poorer performance on speeded tasks [71, 72]. Similarly, impaired respiratory function predicts overall and subcortical brain atrophy as well as white matter hyperintensities [73]. One possible explanation for the finding that MTF was associated with accelerated cognitive decline relative to RTF is that our measures of neurocognitive speed were computer-based reaction time tasks. Performance on these tasks thus reflects not only processing speed, but also motor control and muscle function. Individuals with deficits in mobility function may therefore have been disproportionately impaired on these tasks relative to those with respiratory deficits. Although linked to relevant literature, these explanations are speculative and multiple contributing mechanisms likely account for the frailty-speed associations. Continued research efforts are required in order to understand the pathophysiologic underpinnings of MTF and RTF.

### Strengths and limitations

We acknowledge several methodological strengths and limitations. First, with respect to the former, we used a substantial and well-characterized sample of participants from the VLS. These individuals were tested on three occasions across a 40-year band of aging and were relatively healthy and free of neurodegenerative disease at baseline. These characteristics allowed us to distinguish and subsequently examine the impact of early frailty profiles on normal cognitive aging trajectories. At the same time, our findings may be limited in generalizability to other populations (e.g., more frail older adults; ethnic minorities) or contexts (e.g., continuing care settings). Future investigations should explore this possibility. Second, we examined our research questions using contemporary statistical approaches. Specifically, we derived empirically based frailty profiles using LPA. This data-driven approach boasts several advantages over classical statistical models (e.g., cluster analysis) [11], such as model-based participant classifications, statistical diagnostic tools that elucidate the quality of participant classifications, and information-theoretic indices that favor selection of the most parsimonious model (thus discouraging overfitting). We validated our profiles by examining how they related to the level and rate of change in frailty and neurocognitive speed using the BCH approach, which allowed us to statistically account for misclassification errors. We calculated the primary distal outcome measure using multiple standard neuropsychological tasks, which contributed to a validated, invariant, longitudinal, latent measure of neurocognitive speed. We controlled for the potential confounding effects of age, as well as verified that prediction patterns generalized across sex and could not be attributed to educational background or proportion of deficits accumulated. Third, we assembled baseline data that represented the heterogeneity of frailty. This enabled us to detect nuanced frailty profiles and address a prominent criticism of earlier data-driven research [74]. It is worth noting, however, that our indicators in the LPA do not represent the full range of deficits that older adults may accumulate. For example, due to unavailability, we did not include indicators related to social function (beyond those included in instrumental health) or nutritional status. Previous studies including these indicators did not distinguish social or nutrition profiles [14, 15, 45, 49]. This is a common issue in frailty research. The phenotype approach does not include all possible phenotypes and the frailty index includes no phenotypes, but rather a score that could vary according to the available items. Nevertheless, future studies could explore whether inclusion of social and nutritional deficits may result in profile interpretations and prediction patterns that diverge from the present research.

## Conclusions

Our study distinguished three early frailty profiles using data-driven statistical techniques: not-clinically-frail (NCF), mobility-type frailty (MTF), and respiratory-type frailty (RTF). Whereas the former and larger profile represented older adults with minimal current impairment across multiple indicators of aging morbidity, the latter two profiles represented individuals with marked impairment in either mobility or respiratory function. Prevailing approaches that collapse across markers of aging morbidity may therefore mask important variability, including identification of (a) differentiable profiles that may be characterized as morbidity-intensive portals into broader and chronic frailty and (b) older adults at risk for accelerated cognitive decline and impairment. These profiles were differentially associated with longitudinal change in neurocognitive slowing, such that MTF was associated with the steepest decline, followed by RTF. As new and more effective treatments become available, studies directed towards identifying subgroups of frail older adults who are not yet exhibiting cognitive impairment but who are at increased risk are essential. Our results indicate that older adults presenting with mobility or respiratory complaints may benefit from early and targeted interventions [53, 75]. Future research should explore the extent to which rehabilitation and pharmacologic treatments targeting these deficits may offset or delay cognitive decline and frailty progression.

## Supplementary Information

**Additional file 1.** Supplementary methods, tables, and figures.

## Data Availability

The dataset compiled and analyzed during the current study is available from the corresponding author on reasonable request.

## Abbreviations

AIC: Akaike Information Criterion
BIC: Bayesian Information Criterion
CFI: Comparative fit index
LCA: Latent class analysis
LL: Log-likelihood
LMR-LRT: Adjusted Lo-Mendell-Rubin likelihood ratio test
LPA: Latent profile analysis
MMSE: Mini-mental status exam
MTF: Mobility-type frailty
NCF: Not-clinically-frail
RG: Research goal
RMSEA: Root mean square error of approximation
RTF: Respiratory-type frailty
SABIC: Sample-size adjusted BIC
SRMR: Standardized root mean square residual
VLMR-LRT: Adjusted Vuong-Lo-Mendell-Rubin likelihood ratio test
VLS: Victoria Longitudinal Study
