# Supplementary material for: Portals to frailty? Data-driven analyses detect early frailty profiles
Source: Alzheimers Res Ther. 2021 Jan 4;13:1. doi: 10.1186/s13195-020-00736-w (PMC7780374; doi:10.1186/s13195-020-00736-w)
Supplement: Supplementary file 1 — Additional file 1. Supplementary methods, tables, and figures. [file 13195_2020_736_MOESM1_ESM.docx]

Supplementary Material for: Portals to Frailty? Data-Driven Analyses Detect Early Frailty Profiles

**Methods**

**Measures**

***Simple reaction time****.* Participants were shown a warning stimulus, followed by a signal stimulus, in the middle of a computer screen. Participants were asked to press a response key as quickly as possible upon detecting the signal stimulus. Participants completed 10 practice trials, followed by 50 test trials. Ten randomly arranged trials were presented at each of the 5 intervals separating the warning and signal stimulus (i.e., 500, 625, 750, 875, and 1,000 ms).

***Choice reaction time****.* Participants were presented with a 2 x 2 grid on the computer screen and instructed that one of the plus signs would be replaced by a square (following a 1000 ms delay). Their task was to indicate the location of the square as quickly as possible by pressing the corresponding key on the response console.

***Lexical decision****. ﻿*Participants were shown a string of five to seven letters on the computer screen and informed that their task was to identify, as quickly as possible, whether the letters formed an English word (e.g., *island vs. nabion*). Participants completed 3 practice trials, followed by 60 test trials (30 words and 30 nonwords).

***Sentence verification****.* Participants were presented with a sentence on a computer screen and subsequently asked to determine the plausibility of the sentence as quickly as possible (e.g., *The tree fell to the ground with a loud crash* vs. *The pig gave birth to a litter of kittens this morning*). Participants were administered 4 practice trials, followed by 50 test trials.

***Correction procedures****.* We trimmed extreme outliers from raw latency scores using validated correction procedures [1,2]. Specifically, we applied the following lower and upper limits for each task: (a) simple reaction time: 150 ms lower limit, 2500 ms upper limit, (b) choice reaction time: 150 ms lower limit, 4000 ms upper limit, (c) lexical decision: 400 ms lower limit, 10,000 ms upper limit, and (d) sentence verification: 1000 ms lower limit, 20,000 ms upper limit. We used task-specific lower and upper limits in order to account for variability across tasks in cognitive complexity. We removed trials that fell three standard deviations above or below the sample mean.

**Foundational analyses**

***Confirmatory factor analysis****.* We determined statistical model fit using the following standard indices: (a) χ2 for which a good fit would produce a non-significant result (i.e., *p*  > .05; indicates that the data do not significantly differ from model-based estimates), (b) the comparative fit index (CFI) for which fit is judged by a value of ≥ .95 as good and ≥ .90 as adequate, (c) root mean square error of approximation (RMSEA) for which fit is judged by a value of ≤ .05 as good and ≤ .08 as adequate, and (d) standardized root mean square residual (SRMR) for which fit is judged by a value of ≤ .08 as good [3].

***Longitudinal measurement invariance****.* We tested longitudinal measurement invariance of the speed latent variable by evaluating (a) configural invariance (the same factor loading patterns over time); (b) metric invariance (the same factor loadings over time); and (c) scalar invariance (the same intercepts over time). We tested invariance assumptions by comparing models with unconstrained and constrained parameters using change in CFI, for which changes of < .01 suggest the assumption is reasonable [3].

***Unconditional latent growth modeling****.* In order to establish the functional form of speed, we estimated factor scores and employed these in an unconditional latent growth model. Age was centered at 75 years for the growth model because (a) this is the approximate mean of the 40-year span of data, (b) this as a common inflection point for such age spans in cognitive aging research [4], and (c) this is standard practice in VLS research [2]. We established the best model by testing the following models in sequence (a) a fixed intercept model, which assumes no intra- or inter-individual variability; (b) a random intercept model, which allows for interindividual differences in overall level but assumes no intraindividual change; (c) a random intercept fixed slope model, which allows for interindividual differences in level but assumes that each person changes at a comparable rate; and (d) a random intercept random slope model, which allows for interindividual differences in both level and change [5].

**Table 1**

Complete List of 50 Items Submitted to an Exploratory Factor Analysis

|  | Frailty Item | Coding |
| --- | --- | --- |
| SR | Stroke | 0 = no; 0.33 = yes, not serious; 0.67 = yes, moderately serious; 1 = yes, very serious |
|  | Thyroid condition |  |
|  | Arthritis (rheumatoid and/or osteo-) |  |
|  | Osteoporosis |  |
|  | Cancer |  |
|  | Asthma |  |
|  | Migraines |  |
|  | Stomach ulcer |  |
|  | Kidney or bladder trouble |  |
|  | Gastrointestinal problems (colitis/diverticulitis, gall bladder trouble, and/or liver trouble) |  |
|  | Bronchitis or emphysema |  |
|  | Diabetes |  |
|  | High blood pressure |  |
|  | Sex-related health problems (i.e., gynecological problems or prostate problems) |  |
|  | Anaemia |  |
|  | Drug and/or alcohol dependence |  |
|  | Spinal condition and/or back trouble |  |
|  | Hardening of arteries (i.e., atherosclerosis) |  |
|  | Heart trouble |  |
|  | Other conditions (up to three) |  |
| SR | Number of medications | 0 = 0-3; 0.5 = 4-7; 1 = 8+ |
| SR | Subjective health relative to a perfect state of health | 0 = very good; 0.25 = good; 0.50 = fair; 0.75 = poor; 1 = very poor |
|  | Eyesight relative to age group |  |
|  | Hearing relative to age group |  |
| SR | Health has affected ability to do chores | 0 = no change, improved, N/A; 0.25 = slightly reduced; 0.50 = moderately reduced; 0.75 = drastically reduced; 1 = gave up doing activity |
|  | Health has affected ability to get around town |  |
|  | Health has affected ability to do mental recreational activities |  |
|  | Health has affected ability to do physical recreational activities |  |
|  | Health has affected ability to do hobbies |  |
|  | Health has affected ability to socialize |  |
|  | Health has affected ability to travel |  |
| SR | Stay at home but in chair most of the time | 0 = no; 1 = yes |
| SR | Number of times sick in bed all day in the past year | 0 = 0-3; 1 = 4+ |
| SR | Number of times confined to hospital in the past year | 0 = 0; 0.5 = 1-2; 3+ = 1 |
| SR | Feeling short of breath | 0 = no; 1 = yes |
| SR | Use of a walker, cane, or wheelchair | 0 = no; 1 = yes |
| M | Resting heart rate (bpm) | 0 = 60-99; 1 = < 60 or 100+ |
| M | Pulse pressure (mmHg) | 0 = 32.13-63.90; 0.5 = 64-75.9; 1 = 76+ |
| M | Peak expiratory flow (L/min) | Men: 0 = >340; 1 = ≤340  Women: 0 = >310; 1 = ≤ 310 |
| M | Body mass index (kg/m^2^) | 0 = 18.5-25; 0.5 = 25 to < 30; 1 = < 18.5 or ≥ 30 |
| M | Grip strength (kg) | Men: for BMI ≤ 24, GS ≤ 29; for BMI 24.1-28, GS ≤ 30; for BMI > 28, GS ≤ 32  Women: for BMI ≤ 23, GS ≤ 17; for BMI 23.1-26, GS ≤ 17.3; for BMI 26.1-29, GS ≤ 18; for BMI > 29, GS ≤ 21 |
| M | Timed walk | 0 = ≤10s; 1 = >10s |
| M | Timed turn | 0 = < 90^th^ percentile  1 = within 90^th^ percentile |
| M | Finger dexterity | 0 = < 90^th^ percentile  1 = within 90^th^ percentile |
| SR | CES-D “during the past week, my sleep was restless” | 0 = rarely or none of the time; 0.33 = some or a little of the time; 0.67 = occasionally or a moderate amount of the time; 1 = most or all of the time |
| SR | CES-D “during the past week, I felt depressed” |  |
| SR | CES-D “during the past week, I felt lonely” |  |
| SR | CES-D “during the past week, I could not get going” |  |
| SR | Bradburn negative affect (restless, lonely, bored, depressed, upset due to criticism) | 0 = no to all; 0.2 = yes to one; 0.4 = yes to two; 0.6 = yes to three; 0.8 = yes to four; 1 = yes to all |
| SR | Physical activity at least 2-3 times per week | 0 = yes; 1 = no |

*Note.* SR, self-reported; M, measured; CES-D, Center for Epidemiological Studies Depression Scale.

**Table 2**

Fit Indices for Confirmatory Factor Analysis and Measurement Invariance Testing for Neurocognitive Speed

| Model | AIC | BIC | ﻿χ^2^ | *df* | *p* | RMSEA | CFI | SRMR | ﻿ ﻿ΔCFI |
| --- | --- | --- | --- | --- | --- | --- | --- | --- | --- |
| Configural invariance | 19013.62 | 19268.72 | 217.23 | 33 | <.001 | .09 (.08 - .11) | .96 | .08 | -- |
| Metric invariance | 19010.57 | 19238.82 | 226.187 | 39 | <.001 | .09 (.08 - .10) | .96 | .09 | <.01 |
| Scalar invariance ^a^ | 19022.97 | 19224.36 | 250.58 | 45 | <.001 | .08 (.07 - .09) | .96 | .09 | <.01 |

*Note.* AIC, Akaike information criterion; BIC, Bayesian information criterion; χ2, chi-square test of model fit; *df*, degrees of freedom for model fit; RMSEA, root mean square error of approximation; RMSEA is shown with 90% confidence intervals; CFI, comparative fit index; SRMR, standardized root mean square residual; ﻿ΔCFI = change in CFI.

^a^ Best fitting model.

**Table 3**

Fit Indices for the Unconditional Growth Model for Neurocognitive Speed and the Frailty Index

| Model | (-)2*LL* | npar free | ﻿AIC | *BIC* | *D* | ﻿ ﻿Δ*df* |
| --- | --- | --- | --- | --- | --- | --- |
| Neurocognitive Speed |  |  |  |  |  |  |
| Fixed intercept | 5000.61 | 4 | 5008.61 | 5026.51 | -- | -- |
| Random intercept | 3961.54 | 5 | 3971.54 | 3993.92 | 1039.07^*^ | 1 |
| Random intercept, fixed slope | 3254.56 | 6 | 3266.56 | 3293.41 | 706.98^*^ | 1 |
| Random intercept, random slope ^a^ | 2996.75 | 8 | 3012.75 | 3048.55 | 257.81^*^ | 2 |
| Frailty Index |  |  |  |  |  |  |
| Fixed intercept | -3274.39 | 4 | -3200.11 | -3182.30 | -- | -- |
| Random intercept | -3887.54 | 5 | -3811.64 | -3789.38 | -607.07^*^ | 1 |
| Random intercept, fixed slope | -3836.62 | 6 | -3746.72 | -3720.01 | 69.37 | 1 |
| Random intercept, random slope ^a^ | -4109.26 | 8 | -4015.40 | -3979.78 | -259.78^*^ | 2 |

*Note.* -2*LL*, -2 log-likelihood; npar, number of parameters; AIC, Akaike information criterion; BIC, Bayesian information criterion; *D*, difference statistic.

^a^ Best fitting model.

^*^ *p* < .001

**Table 4**

Model Estimated Class-Specific Means and Standardized Mean Differences for Each Indicator

|  | Not-clinically-frail | Mobility-type | Respiratory-type | | Standardized mean differences | | |
| --- | --- | --- | --- | --- | --- | --- | --- |
| Indicator | 542 (84%) | 59 (9%) | 48 (7%) | MTF - NCF | | RTF - NCF | MTF - RTF |
| Physical activity | 0.16 (0.16) | 0.29 (0.20) | 0.27 (0.22) | 0.75 | | 0.67 | 0.11 |
| Cardiac symptoms | 0.07 (0.10) | 0.17 (0.17) | 0.10 (0.11) | 0.86 | | 0.24 | 0.78 |
| Respiratory symptoms | 0.03 (0.05) | 0.03 (0.08) | 0.46 (0.13) | 0.14 | | **6.96** | **-4.72** |
| Comorbidity | 0.12 (0.14) | 0.12 (0.14) | 0.19 (0.21) | 0.01 | | 0.56 | -0.52 |
| Emotional well-being | 0.19 (0.18) | 0.16 (0.14) | 0.24 (0.18) | -0.14 | | 0.28 | -0.55 |
| Instrumental health | 0.06 (0.10) | 0.20 (0.17) | 0.12 (0.13) | 1.31 | | 0.60 | 0.80 |
| Mobility | 0.05 (0.10) | 0.58 (0.14) | 0.12 (0.17) | **5.09** | | 0.61 | **3.90** |

*Note.* Results presented as mean (standard deviation). Indicators are coded such that higher scores denote greater impairment. Bolded values represent indicators with a high degree of class separation. NCF, not-clinically-frail; MTF, mobility-type frailty; RTF, respiratory-type frailty.


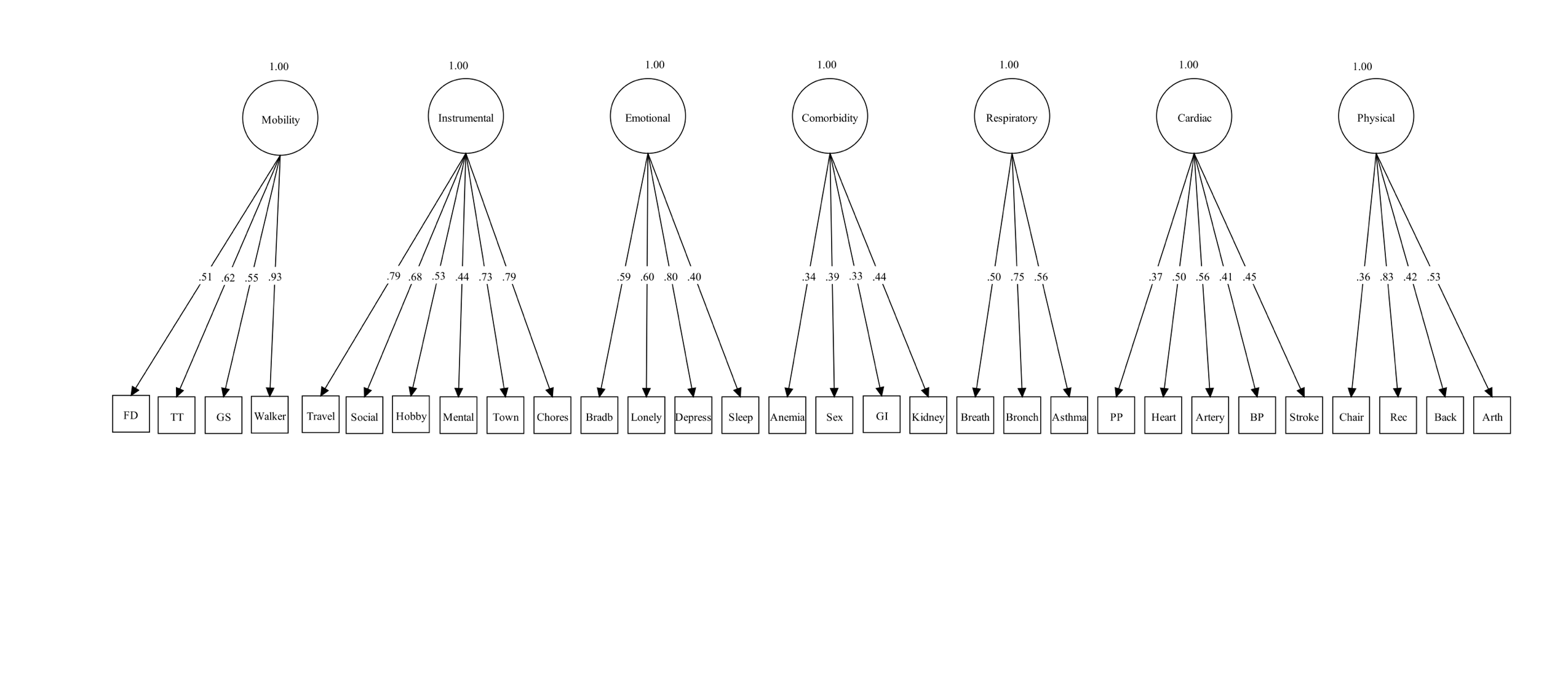


**Fig. 1** Confirmatory factor analysis model of multi-morbidity data.

*Note.* FD, finger dexterity; TT, timed turn; GS, grip strength; Walker, use of a walker, cane, or wheelchair; Travel, health has affected ability to travel; Social, health has affected ability to socialize; Hobby, health has affected ability to do hobbies; Mental, health has affected ability to do mental recreational activities; Town, health has affected ability to get around town; Chores, health has affected ability to do chores; Bradb, Bradburn negative affect; Lonely, during the past week I felt lonely; Depress, during the past week I felt depressed; Sleep, during the past week my sleep was restless; Sex, sex-related health problems; GI, gastrointestinal problems; Kidney, kidney or bladder trouble; Breath, feeling short of breath; Bronch, bronchitis or emphysema; PP, pulse pressure; Heart, heart trouble; Artery, hardening of arteries; BP, high blood pressure; Chair, stay at home but in chair most of the time; Rec, health has affected ability to do physical recreational activities; Back, spinal condition and/or back problems; Arth, arthritis. Standardized factor loadings are shown. All loadings were significant at *p* < .05. Covariances and residuals are not depicted. Response scales for each item are outlined in Table 1.

References

1. Dixon RA, Garrett DD, Lentz TL, MacDonald SWS, Strauss E, Hultsch DF. Neurocognitive markers of cognitive impairment: exploring the roles of speed and inconsistency. Neuropsychology. 2007;21(3):381–99.

2. McFall GP, Wiebe SA, Vergote D, Anstey KJ, Dixon RA. Alzheimer’s genetic risk intensifies neurocognitive slowing associated with diabetes in nondemented older adults. Alzheimer’s Dement (Amst). 2015;1:395–402.

3. Little TD. Longitudinal structural equation modeling. New York: NY: Guilford Press; 2013.

4. Dixon RA, Small BJ, MacDonald SWS, McArdle JJ. Yes, memory declines with aging-- but when, how, and why? In: Naveh-Benjamin M, Ohta N, editors. Memory and aging: current issues and future directions. New York, NY: Psychology Press; 2012. p. 325–347.

5. Singer JD, Willett J. Applied longitudinal data analysis: modeling change and event occurrence. New York, NY: Oxford University Press; 2003.
